# Choline Kinase α Inhibitors MN58b and RSM932A Enhances the Antitumor Response to Cisplatin in Lung Tumor Cells

**DOI:** 10.3390/pharmaceutics14061143

**Published:** 2022-05-27

**Authors:** Juan Carlos Lacal, Rosario Perona, Javier de Castro, Arancha Cebrián

**Affiliations:** 1 Instituto de Investigaciones Biomédicas, CSIC/UAM, 28029 Madrid, Spain; rperona@iib.uam.es (R.P.); arancha.cebrian@quironsalud.es (A.C.); 2 Instituto de Investigación Sanitaria Hospital La Paz, IDIPAZ, 28046 Madrid, Spain; javier.decastro@salud.madrid.org; 3 Instituto de Investigación Sanitara Fundación Jiménez Díaz, 28040 Madrid, Spain

**Keywords:** choline kinase, lipid metabolism, cisplatin, lung tumors, combinatorial chemotherapy

## Abstract

Lung cancer is one of the main causes of death in developed countries, and non-small cell lung cancer (NSCLC) is the most frequent type (80% of patients). In advanced NSCLC, platinum-based chemotherapy is the frontline palliative treatment, but less than 5% of patients achieve prolonged survival. Immunotherapy has recently been proposed as the standard of care (SoC) as either monotherapy or in combination with chemotherapy for advanced NSCLC. The levels of expression of PD-L1 are the only predictive biomarkers for patient assessment. Although around 30% of patients receiving immunotherapy achieve 5-year survival, a significant number does not benefit from this novel therapeutic approach. Therefore, there is a need for novel strategies to improve clinical outcomes. The expression level of choline kinase α (ChoKα) is increased in a large number of human tumors, including NSCLC tumors, and constitutes an independent prognostic factor for early-stage NSCLC patients. Thus, ChoKα has been postulated as a new target drug in cancer therapy. The combination of cisplatin with novel targeted drugs such as choline kinase inhibitors may improve both the survival rates and the quality of life of NSCLC patients and may serve as the basis for the development of new therapeutic approaches. To that aim, we developed several in vitro and in vivo approaches to assess the antitumor activity of a novel combination regimen using cisplatin and ChoKα inhibitors. Our results suggest that a proper combination of specific inhibitors of the NSCLC prognostic factor ChoKα and platinum-based conventional chemotherapy might constitute a new, efficient treatment approach for NSCLC patients. This novel approach may help reduce the toxicity profile associated with cisplatin since, despite the advances in NSCLC management in recent years, the overall 5-year survival rate is still poor.

## 1. Introduction

Over 2.2 million cases of lung cancer are diagnosed each year worldwide, constituting the leading cause of cancer-related mortality, with 1.8 million deaths in 2020 (Globocan, 2020; see https://gco.iarc.fr/today/online-analysis-table?v=2020&mode=cancer&mode_population=continents&population=900&populations=900&key=asr&sex=0&cancer=39&type=1&statistic=5&prevalence=0&population_group=0&ages_group%5B%5D=0&ages_group%5B%5D=17&group_cancer=1&include_nmsc=0&include_nmsc_other=1, accessed on 23 May 2022). Over 80% of all lung cancers belong to the “non-small cell” histological subgroup, known as non-small cell lung cancer (NSCLC). A total of 20–25% of NSCLC patients present with stage III, and 35–40% are stage IV. For this advanced disease, firstline treatment options have evolved in recent years with immunotherapy in patients with high levels of PD-L1. This approach has rendered an impressive 30% survival rate at 5 years; however, the remaining 70% experience no benefit, and the mainstay still consists of platinum-based chemotherapy doublets. Furthermore, concurrent chemoradiotherapy is still the therapeutic approach used in patients with unresectable stage III and good performance status. However, a wide range of side effects are associated with this regimen, including nausea, vomiting, and myelosuppression, as well as oto-, neuro-, and nephro-toxicity, accounting for a strong decrease in quality of life [1,2].

In order to reduce the toxicity profile of cisplatinum-based regimens, the use of new generations of platinum drugs, including carboplatin and oxaliplatin, has been introduced in the backbone of systemic chemotherapy in NSCLC, but adverse events still display a relevant impact on the quality of life of these patients. Subsequently, the introduction of third-generation cytotoxic drugs to platinum agents (known as doublets), including paclitaxel, docetaxel, gemcitabine, pemetrexed, and vinorelbine, has yielded higher response rates, maintaining the use of platinum doublets as standard therapy for NSCLC treatment [3,4].

More recently, new targeted, personalized therapies based on identified oncogenic drivers usually alone, but also combined with platinum doublets, with the introduction of patient selection procedures, have been explored with the main goals of improving clinical outcomes and minimizing side effects of treatments within drug optimization. Among these, interferences with EGFR, ALK, ROS 1, BRAF, NTRK, RET, and MET signaling have provided improved outcome in these patients [5]. Finally, the advent of immunotherapy based on specific antibodies against the programmed death (PD-1) receptor, the programmed death-ligand 1 (PD-L1), and the cytotoxic T-lymphocyte–associated protein 4 receptor (CTLA-4) has had a great impact on survival for some NSCLC patients. In metastatic tumors, recent clinical trials favored the use of these immune checkpoint inhibitors (ICIs) as first- or second-line treatments alone or in combination with chemotherapy [6]. The levels of expression of PD-L1 in tumor cells are the reference biomarkers for patient assessment of this approach. However, despite these advances, a significant number of patients cannot benefit from these therapies. Therefore, there is still a need for testing new targets and new combinatorial regimens.

Choline kinase alpha (ChoKα), the first enzyme of the Kennedy pathway for the biosynthesis of the major phospholipid of the plasma membrane, phosphatidylcholine (PC), has been described as a novel oncogene [7] whose gene expression levels constitute a new prognostic factor in NSCLC patients [8]. Consequently, ChoKα-specific inhibitors have been designed and showed strong in vitro antiproliferative activity against human lung-cancer-derived models, as well as efficient antitumor activity in vivo in nude mice against different human xenografts [9]. These results were further confirmed using siRNA-driven interference molecules [10,11,12]. The development of RSM-932A/TCD-717, a highly specific ChoKα inhibitor [13] allowed entrance into a Phase I clinical trial as a new “first in class” targeted therapy (http://clinicaltrials.gov/ct2/show/NCT01215864; accessed on 6 April 2022). Here, we present in vitro and in vivo approaches to assess the antitumor activity of a novel combination regimen using cisplatin and ChoKα inhibitors.

## 2. Materials and Methods

### 2.1. Patients

Specimens of lung cancer tissue from 63 randomly selected patients who underwent surgical resection for NSCLC between 2001 and 2004 and who were followed up on by the Medical Oncology division at La Paz Hospital in Madrid were used for this analysis. No adjuvant therapy was administrated to these patients. The institutional review board of the hospital approved the study, and written informed consent was obtained from all the patients.

### 2.2. Primary Cultures of NSCLC Tumors

The resected tissues from the NSCLC patients were enzymatically and chemically dissociated (Cell dissociation sieve-tissue grinder Kit, SIGMA-Aldrich, Madrid, Spain), and the obtained cells were seeded into 96-well plates (BD, Falcon, Bioscience, San Jose, CA, USA), as previously described [14]. The cells were treated with increasing concentrations (0, 0.5, 1, 5, 10, and 20 µM) of cDDP, taxol, vinolrelbine, gemcitabine, or MN58b for 10 days in DMEM:F12HAM (Ref:D8437, SIGMA-Aldrich) supplemented with 10% fetal bovine serum (FBS) (Life Technologies, Grand Island, NY, USA). The final persistent population in each well was quantified with an Alamar blue assay, as previously described [14]. Alamar blue was added directly into the culture media at a final concentration of 10%, and the plate was returned to the incubator. The optical density of the plate was measured at 570 and 600 nm with a standard spectrophotometer at 3 h after adding Alamar blue. The cell viability was calculated according to the manufacturer’s protocol (Biosource Europe, Nivelles, Belgium).

### 2.3. Cell Lines and Chemicals

All the cell lines used in this study were maintained under standard conditions of temperature (37 °C), humidity (95%), and carbon dioxide (5%). Epithelial non-small lung cancer H460 cells (generated from a pleural effusion of a patient with large cell lung cancer) and H1299 cells (derived from an NSCLC carcinoma) were maintained in RPMI supplemented with 10% fetal bovine serum (FBS) (Life Technologies, Grand Island, NY, USA). Cell lines resistant to the ChoKα-specific inhibitors MN58b or RSM-932A/TCD-717 were generated by prolonged continuous exposure to increasing concentrations of each drug and were maintained in RPMI supplemented with 10% FBS. A parallel control (H460 stock) of the cell line in the absence of the compounds was kept in culture for the same time. In-house-generated MN58b and RSM-932A/TCD-717 were dissolved in PBS and DMSO: PBS 2:1, respectively. Cisplatin was obtained from Ferrer Farma (EGF speciality).

### 2.4. Cell Proliferation Assays and Combined Index Evaluation

An amount of 6000 cells/well was seeded into 96-well flat-bottomed plates (BD, Falcon, Bioscience, San Jose, CA, USA) and incubated for 24 h under standard conditions. The cells were then treated with different concentrations of ChoKα inhibitors and cisplatin either concomitantly or in a sequential manner, with cisplatin for 5 h followed by a ChoKα inhibitor (MN58b or RSM-932A/TCD-717) for the next 40 h. Quantification of the number of cells remaining in each well was carried out using the MTT (3-(4,5-dimethylthiazol-2-yl)-2,5-diphenyltetrazolium bromide) method. The absorbance was read at 560 nm with a VersaMax Microplate Reader (Molecular Devices, Sunnyvale, CA, USA). Drug interaction between the ChoKα inhibitors and cisplatin was assessed using a combination index (CI) [15] where CI < 1, CI = 1, and CI > 1 indicated synergistic, additive, and antagonistic effects, respectively. The CI value was calculated as CI = D_1_/Df_1_ + D_2_/Df_2_, where Df_1_ and Df_2_ are the concentrations of MN58b or RSM-932A/TCD-717 and cisplatin, respectively, required to inhibit cell growth by 50%, and D_1_ and D_2_ are the drugs’ concentrations in the combination treatment that also inhibited cell growth by 50%. The data analysis was performed with Calcusyn software (Biosoft, Oxford, UK).

### 2.5. Flow Cytometric Assay

The cell cycle distribution was determined by DNA content analysis after propidium iodide staining. The cells were treated with ChoKα inhibitors and cisplatin alone or in combination for 45 h. The cells were then harvested, stained, and incubated with 50 µL detergent and 1 mL IP (50 µg/mL) containing RNase (20 µg/mL). The DNA content of approximately 4 × 10^5^ stained cells was analyzed using a Coulter XL-MZL flow cytometer. The fractions of cells in apoptosis, G0-G1, S, and G2-M phases were analyzed with DNA program software.

### 2.6. Western Blot Analysis

The cells were incubated with ChoKα inhibitors and cisplatin alone or in combination. Equal amounts of protein (30 μg) were loaded into 15% SDS-PAGE acrylamide gels, and the resolved proteins were transferred onto nitrocellulose membranes. The following antibodies were used: anti-p-JNK (Promega, v7932), anti-p-p38 (Cell Signaling 9211S), and anti-P38 (Santa Cruz sc-535), with anti-α-tubulin (Sigma T9026) as a load control.

### 2.7. In Vivo Antitumoral Assays

Female athymic BALB/C nude mice were supplied by Jackson. The animal care and surgery protocols were approved by the Animal Care Committee. The mice were maintained with autoclaved water and sterile food ad libitum. The mice were inoculated subcutaneously with injections of 1 × 10^6^ H460 cells in the flank of each mouse mixed 1:1 with matrigel (354234, BD Bioscience, Haryana, India). The tumor sizes were determined using micrometer calipers, and when the size of the tumors was approximately 0.1 cm^3^, the mice were divided into 8 different groups: control (vehicle); 2 mg MN58b/kg for 3 days/week; 1 mg cisplatin/kg for 2 days/week; 2 mg RSM-932A/TCD-717/kg for 2 days/week; and MN58b-cisplatin or RSM-932A/TCD-717-cisplatin combinations following both a sequential and concomitant schedule. MN58b and cisplatin were dissolved in PBS and injected i.p. in the amount of 0.1 mL/mouse. RSM-932A/TCD-717 was dissolved in DMSO:PBS 2:1 and diluted with PBS to give appropriate concentrations. The tumor sizes were measured twice a week at their greatest lengths and widths, and the volumes were calculated as (tumor width^2^ × tumor length)/2.

### 2.8. Statistical Analysis

**Figure 1:**
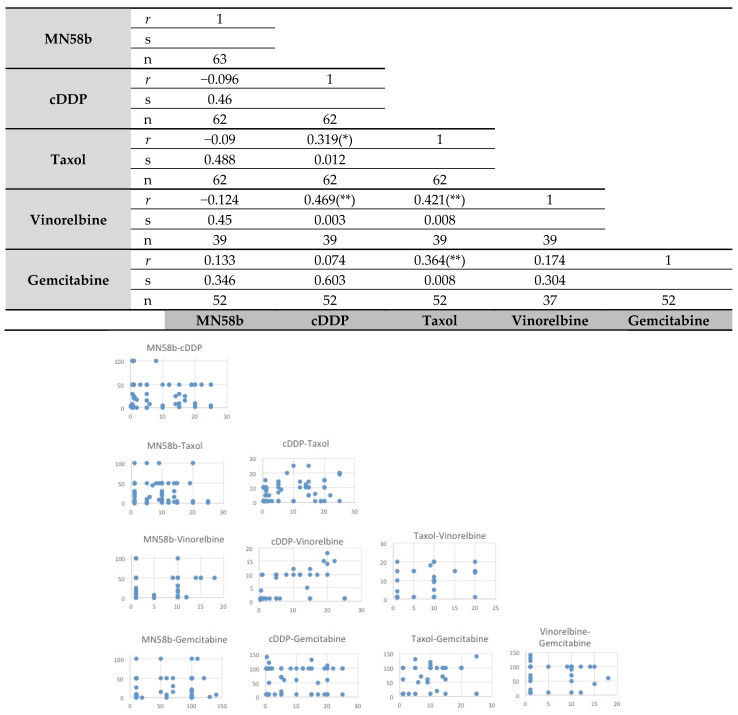
Pearson’s correlation analysis of intrinsic resistance of primary tumors of patients with NSCLC to different antitumoral agents. Upper panel: the statistical analysis was performed as indicated in Material and Methods for the response rate of the samples to the different treatments. MN58b was the only drug whose response did not correlate in responsiveness to any other agent. *r*: Pearson’s correlations coefficient; s: significant correlation, bilateral (* *p* ≤ 0.05; ** *p* ≤ 0.01); n: number of primary tumors analyzed. Lower panel: dispersion graphs for each pair of drugs.

The correlations in responsiveness to the different treatments in the tumor samples of patients with NSCLC were performed following Pearson’s correlations (*r*) to measure the linear correlation between two different treatments. This is achieved by determining the ratio between their covariance and the product of their standard deviations according to the following formula:

*r*_x,y_ = _COV (x,y)_/σ_x_σ_y_

where _COV_ is the covariance, σ_x_ is the standard deviation of x, and σ_y_ is the standard deviation of y. This rendered a normalized measurement of the covariance, such that the result always had a value between −1 and +1. A Pearson’s correlation of (*r* = −1) meant a negative perfect correlation, while (*r* = 1) was a perfect positive alignment. A value of 0 implied that there was no linear dependency between the variables. The values for *r* represented comparisons between pairs of the drugs used in the study and are reflected in Figure 1. The dispersion graphics are also reported.

Comparisons in the tumor volumes between the untreated and treated groups were performed using a nonparametric Mann–Whitney test. Two-sided *p*-values less than 0.05 were considered statistically significant. All the calculations were performed using SPSS software, version 20.0 (SPSS Inc., Chicago, IL, USA).

## 3. Results

### 3.1. Cisplatin-Intrinsic-Resistant NSCLC Tumors Are Sensitive to ChoKα Inhibition

The primary cultures from 63 resected NSCLC tumors were established and cultivated for 10 days, during which they were treated with increasing concentrations of the ChoKα-specific inhibitor MN58b, cisplatin (cDDP), taxol, vinorelbine, or gemcitabine. Considering resistance when nearly 100% of the cells remained alive for the maximum concentration of the drug at day 10, different resistance rates to the treatments were found. As shown in Table 1, more than 50% of the samples were resistant independently to the treatment, in keeping with the lack of response to chemotherapy observed in the clinic.

A correlation analysis of the responsiveness of these tumors to the different treatments was then performed (Figure 1). Pearson’s correlation indicated significant cross-resistance among cisplatin, taxol, vinorelbine, and gemcitabine. By contrast, MN58b showed no significant cross-resistance to any of the chemotherapeutic drugs tested, consistent with its different mechanism of action [16,17].

To further confirm the absence of cross-resistance between ChoKα inhibitors and cisplatin, resistant H460 cells were generated by exposure to increasing doses of MN58b or RSM-932A/TCD717. The cell lines acquired drug resistance over a long-term period of 9–10 months, after which their sensitivity to both ChoKα inhibitors and cisplatin was determined (Table 2). The fold resistance was from 7–8 for RSM-932A/TCD-717 and from 49–73 for MN58b. A control cell line (H460 stock) was maintained in culture for the same time without treatment and was included as a comparison with the parental cell line. As a consequence of the similar mechanisms of action of the two ChoKα inhibitors, resistant cells to either MN58b (H460 MN58R) or to RSM-932A/TCD-717 (H460 TCD717R) showed an important cross-resistance between them. By contrast and consistent with a different mechanism of resistance acquisition, a significant lack of cross-resistance was found with cisplatin in both cases. In fact, cells resistant to the ChoKα inhibitors were slightly more sensitive to cisplatin.

### 3.2. Synergism of Cisplatin and ChoKα Inhibitors against NSCLC Cells

**Figure 2:**
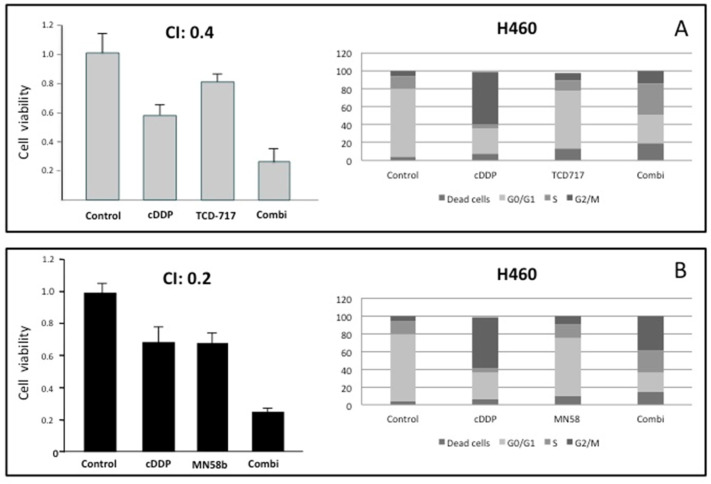

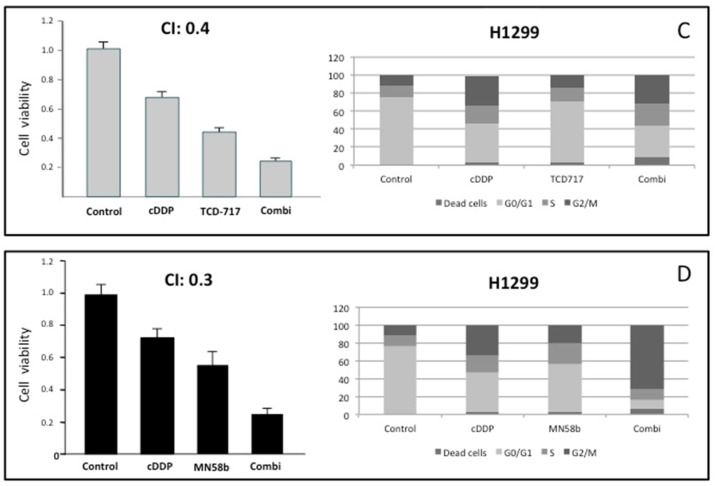
Synergistic effect of ChoKα inhibitors and cisplatin in H460 and H1299 cells. (**A**,**B**) H460 cells were exposed to cisplatin for 5 h. Then, cells were treated with RSM-932A/TCD-717 (**A**) or MN58b (**B**) for 40 h. Cytotoxicity was evaluated by MTT assay (left panels). Combinations of the two drugs was also tested using flow cytometry analysis (right panels). (**C**,**D**) H1299 cells were treated as indicated for H460 cells with either RSM932A/TCD-717 (**C**) or MN58b (**D**). Represented CI value in each case is the mean of three independent experiments for each concentration in quadruplicate. As shown for both drugs, a CI < 1 indicated a strong synergistic effect. Furthermore, combination of the two drugs increased cell death compared to the two drugs alone, as determined by flow cytometry analysis. As shown, similar results were obtained with both cell lines.

The results shown above suggest the putative effectiveness of combined therapy between cisplatin and ChoKα inhibitors. In order to address this issue, the cytotoxic effects of combinations of cisplatin and the ChoKα inhibitors MN58b or RSM-932A/TCD-717 were determined in the human NSCLC H460 cell line (Figure 2A,B). To this end, cell viability was determined after treatment with different drug concentrations and schedules. Strong synergism (combination index, CI ≤ 0.4) was observed when the cells were treated following a sequential treatment initiated with cisplatin for a short period of time (5 h), followed by treatment with RSM-932A/TCD-717 (Figure 2A) or MN58b (Figure 2B) for longer times. Synergism was observed in the different timepoints analyzed, treating with ChoKα inhibitors from 24 to 72 h. A moderate synergistic effect was also found with both agents following a concomitant treatment (CI = 0.5–0.8) (data not shown). These results were confirmed using a second NSCLC cell line, H1299 (Figure 2C,D).

With the aim of further validating these results, we investigated the effect of this combination on cell cycle distribution using flow cytometry analysis. As shown in Figure 2, the combination of these agents displayed a stronger antiproliferative effect than when treating with these drugs separately. Furthermore, the promotion of cell death mediated by the combination was observed even under conditions when these agents separately displayed a minimum effect.

**Figure 3:**
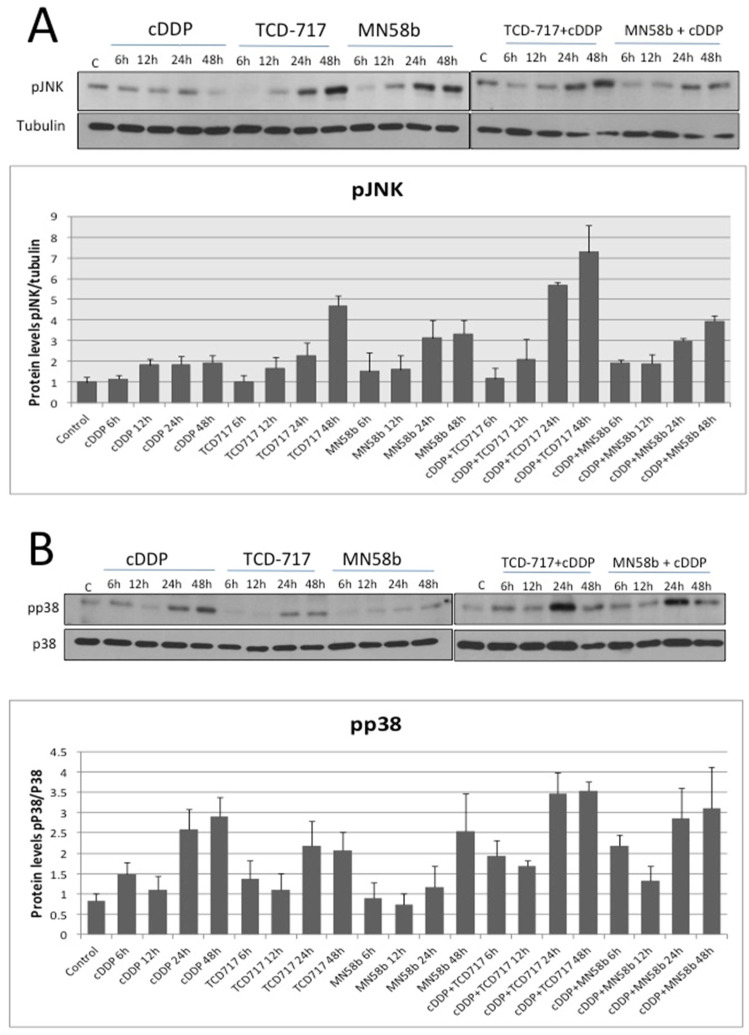
MAPK pathway activation in response to treatment with cisplatin combined with ChoKα inhibitors. H460 human NSCLC cells were exposed to cisplatin, ChoKα inhibitors, or both treatments following a sequential schedule and showing a synergistic effect (cisplatin followed by ChoKα inhibitors). p-JNK (**A**) and p-p38 (**B**) levels were analyzed by Western blotting at different timepoints. Tubulin or total p38 protein were used to normalize. Results are the means of three independent experiments. Western blots are representative of one single experiment.

With the aim of elucidating the mechanism of this synergism, the putative modulation mediated by ChoKα inhibitors of JNK and p38 known to be involved in cell death induced by cisplatin [18] was investigated. As shown in Figure 3A, a two-fold activation of JNK was promoted by cisplatin after 12 h of treatment. ChoKα inhibitors induced a significant greater increase of activation of this pathway (3–4 fold). However, the major effect was observed after the combination of cisplatin and RSM-932A/TCD-717, increasing basal levels of p-JNK up to seven-fold induction. On the other hand, the p38 pathway was significantly more affected by cisplatin than by the ChoKα inhibitors (Figure 3B), also with greater effects after combination. These results suggested that the p38 pathway is mainly activated by cisplatin, whereas JNK is mostly modulated by the ChoKα inhibitors, suggesting that the synergistic effect may be a consequence of a complementary increase in both pathways of the combinatory regimen.

### 3.3. ChoKα Inhibitors Potentiate the Antitumoral Efficacy of Cisplatin In Vivo against NSCLC Xenografts, Reducing the Toxicity Associated with Cisplatin Treatment

**Figure 4:**
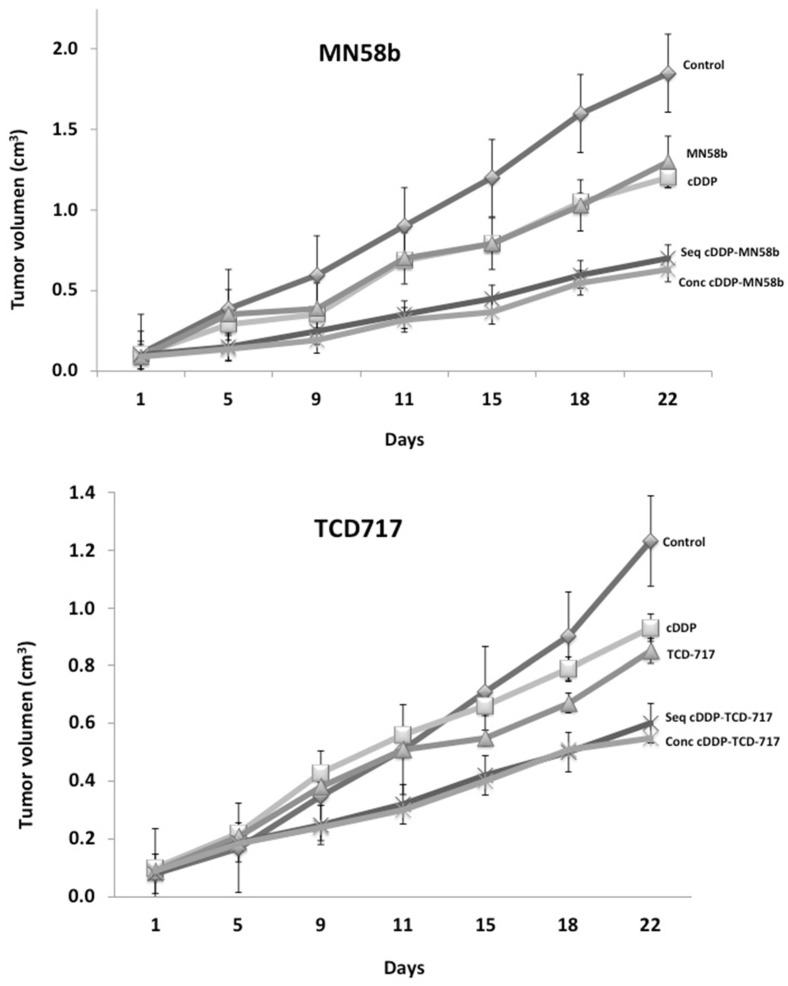
Tumor growth inhibition on H460 xenografts by combination of MN58b or RSM-932A/TCD-717 and cisplatin. H460 cells were injected in nude mice, and when tumor volumes reached 0.1 cm^3^, animals were randomly distributed in groups (*n* = 8) to start treatments. Mice were treated with the indicated ChoKα and cisplatin (cDPP) either alone or following sequential or concomitant schedules. A significant synergistic effect was observed with the combined therapy in both cases: MN58b (upper) and RSM-932A/TCD-717 (lower).

Cisplatin is still used in firstline chemotherapeutic regimens for NSCLC patients. As shown above, synergistic effects were observed when this agent was combined with ChoKα inhibitors in vitro in NSCLC cells. In order to further evaluate the potential therapeutic effect of these combinations, a series of experiments was carried out in vivo using human NSCLC H460 xenografts. Mice were inoculated subcutaneously with an injection of 1 × 10^6^ H460 cells in the flank mixed with Matrigel, as described in Material and Methods. When tumor volumes reached 0.1 cm^3^, the animals were randomly distributed in groups (*n* = 8), and treatments were assayed according to the in vitro regimens. The schedules of the treatments were determined when the maximum efficacy was reached following sequential treatment regimes initiated by cisplatin. A schematic representation of the followed treatments is shown in Table 3. Treatment of the NSCLC H460 tumors with ChoKα inhibitors in combination with cisplatin resulted in a significant reduction in tumor growth compared to these drugs alone (Figure 4).

With this combination therapy, no external signs of toxicity were observed during the treatment, as indicated by the maintenance of body weight. The concomitant treatment of MN58b reached the same efficacy as that of the sequential group, but much higher doses of cytotoxic agents were required. By contrast, to obtain similar effectiveness in the inhibition of tumor growth (65%) using cisplatin as a single agent, its dosage had to be increased from 1 to 4 mg/kg, conditions in which clear signs of toxicity were observed, including a significant reduction in body weight (Table 3). Similar results were obtained when the RSM-932A/TCD-717 inhibitor was used (Table 3).

## 4. Discussion

NSCLC accounts for 80–85% of all bronchogenic malignancies. Included in this category are squamous cell carcinoma (25%), adenocarcinoma (40%), and large cell carcinoma (10%). Extensive research has been performed in the last decade using newly developed drugs as an important attempt to improve clinical outcomes, aiming at achieving higher responses and longer survival rates. In this sense, although the introduction of new, active agents, including paclitaxel, docetaxel, gemcitabine, pemetrexed, and vinorelbine, was promising, the finding of strong cross-resistances is a serious drawback [4]. Furthermore, cancer embraces different diseases, and specific drugs and regimens are required for each specific tumor type. Currently, among the different drugs approved by the FDA as anticancer agents, only a few are related to NSCLC. Bevacizumab, a monoclonal antibody that binds to the vascular endothelial growth factor (VEGF), blocking interaction with its receptors on endothelial cells, prevents the initiation of new blood vessel growth. Some studies have demonstrated that the addition of bevacizumab to platinum-based chemotherapy in addition to gemcitabine or paclitaxel improves chemotherapy response, progression-free survival, and overall survival. The use of the tyrosine kinase inhibitor gefitinib first and, more recently, osimertinib proved to be efficient as a first therapy for NSCLC habouring EGFR mutations [5].

Efforts have been dedicated in recent years to immunotherapy based on specific antibodies against PD-1, its ligand (PD-L1), and CTLA-4. This effort has translated into improved survival for NSCLC patients. Both first- and second-line treatments have been intensively explored, either alone or in combination with diverse chemotherapy treatments. As a result of this search for improved protocols and regimens, the use of these ICIs along with chemotherapy seems to be most promising and has translated into improved survival for NSCLC patients [6]. However, for 70% of these NSCLC patients, the OS is still in very poor figures and makes it necessary to investigate further alternative targets and new combinatorial regimens. Keeping with this, the use of drugs with different mechanisms of action may be useful for overcoming drug resistance to individual treatments. Here, we reported the successful combination of platinum-based chemotherapy with a novel targeted therapy approach as an innovative strategy against solid tumors, such as inhibition of an enzyme that is critical for phospholipid metabolism.

Both primary and acquired resistance is one of the major problems in cancer treatment. The mechanism of action for cisplatin consists of the interaction with N7 sites of purine bases to form inter- and intrastrand crosslinks. The main mechanisms of resistance to cisplatin are reduction in the accumulation of cisplatin by changing uptake or efflux; inactivation of the drug by glutathione, metallothionein, or other sulphur-containig molecules; increased repair of adducts; and increased cisplatin adduct tolerance [19,20,21,22,23]. In contrast, the mechanism of action for ChoKα has been related to the metabolic disbalance produced by the inhibition of phosphatdylcholine synthesis. The inhibition of this enzyme by pharmacological or siRNA approaches produces a loss of mitochondrial potential and cytochrome c release, increased ceramide production, ER stress, unfold protein response (UPR), and ROS homeostasis via glutathione levels. Additional effects due to mitochondria function render a reduction in citrate synthase expression and AMPK activation. Finally, this cascade of events induces an increase in glucose and acetate uptake to overcome the metabolic stress. All these events induce apoptosis or necrosis, specifically in cancer cells (reviewed in [9]).

Resistance to ChoKα has been related to an increase in acid ceramidase, which drains the elevated levels of ceramides and allows cancer cells to keep proliferating [16]. This effect was found in primary NSCLC cultures and in ChoKα-resistant H460 cells, and it was reverted by acid ceramidase inhibitors [24]. However further studies are needed to define precisely a complete set of biomarkers to define ChoKα inhibitor resistance. This will allow efficient selection for NSCLC patients who are potentially resistant to the proposed combination.

In this study, we demonstrated that neither cisplatin nor other drugs usually taking part in platinum-based chemotherapy of NSCLC patients displayed cross-resistance with ChoKα inhibitors. These results may be explained by the differential mechanisms of action of these novel drugs, whose activities rely on inhibiting ChoKα, the first enzyme of the biosynthesis of the major phospholipid component of cell membranes [16,17], or interfering with the replication of DNA [20]. This effect is reflected in the responsiveness of primary tumors from NSCLC patients, and also in tumor-derived cell lines [24]. Therefore, when cells are promoted to division, they are simultaneously blocked at two different phases of the cell cycle, increasing the possibilities to overcome resistance to any of these agents when used alone. This is further supported by the evidence presented in our study where the p38 and the JNK pathways were differentially activated by cisplatin and ChoKα inhibitors, while the combination of both drugs triggered a complementary increase in both pathways.

We also demonstrated here, both in vitro and in vivo, that the use of this combined therapy achieved another important aim of a combination regimen: obtaining similar efficacy using lower concentrations of the chemotherapeutic agents and, therefore, reducing their associated toxicities. ChoKα inhibitors have entered Phase I clinical trials, in part based on the involvement of this enzyme and the effect of its inhibitors on NSCLC. In this sense, ChoKα gene expression levels are independent prognosis factors for early-stage NSCLC patients [8], suggesting an easy molecular method for identifying patients that could benefit from this treatment. Therefore, since platinum-based chemotherapy continues to constitute a key tool in the management of NSCLC and ChoKα inhibition constitutes a “first in class” targeted therapy for NSCLC patients, our study provides the basis for a promising new alternative of combination treatment therapy for patients with this disease.

## 5. Conclusions

Cancer is a multifactorial disease with a large diversity of clinical manifestations. Lung cancer represents one the most devastating types of cancer, being responsible for over 1.8 million deaths every year worldwide. NSCLC represents over 80% of all lung cancers. Significant improvements in clinical management have been achieved in the last decade with the introduction of third-generation cytotoxic drugs, targeted personalized therapies, and immune checkpoint inhibitors (ICIs). However, the clinical outcome of a 5-year survival rate is still poor. New therapeutic approaches are urgently needed for these patients. We provided evidence that ChoKα is a bona fide new target for NSCLC patients and that its inhibitors are potent therapeutic agents that can be efficiently combined with cisplatin-based chemotherapeutic regimens. A limitation of our study is that we presented this effect in two established NSCLC cell lines, H460 (derived from a large cell lung cancer) and H1299 (derived from a carcinoma), which represent only 50% of the NSCLC types. This novel strategy requires confirmation in appropriate Phase II clinical trials.

## Figures and Tables

**Table 1:** Responsiveness of NSCLC primary cultures to different antitumor agents. Resected tissues from NSCLC patients were dissociated to obtain primary cultures that were treated for 10 days with different concentrations of the indicated drugs. Cell viability was determined by Alamar blue. The number of total samples examined in each case and the number of samples sensitive or resistant to each treatment are reported with their proportions (%).

Drug	NSCLC Samples	Sensitive (%)	Resistant (%)
**MN58b**	63	35 (55.6)	28 (44.4)
**cDDP**	62	31 (50.0)	31 (50.0)
**Taxol**	62	27 (43.5)	35 (56.5)
**Vinorelbine**	39	15 (38.5)	24 (61.5)
**Gemcitabine**	52	18 (34.6)	34 (65.4)

**Table 2:** Absence of cross-resistance between cisplatin and ChoKα inhibitors. NSCLC H460 cells and H460 cells made resistant to ChoKα inhibitors MN58b (H460 MN58R) or RSM-932A/TCD-717 (H460 TCD717R) were treated with different concentrations of MN58b, RSM-932A/TCD-717, and cisplatin. The concentration where 50% of the cell proliferation was inhibited (IC_50_) was determined by MTT method. Data represent the mean ± SD of 3 to 9 independent experiments, each performed in quadruplicate. The fold induction of resistance is shown within parentheses.

Cell Line	IC_50_ MN58b (μM)	IC_50_ TCD-717 (μM)	IC_50_ cDDP (μM)
**H460**	0.28 ± 0.12	1.11 ± 0.4	16.60 ± 1.8
**H460 Stoc**	0.39 ± 0.19	1.32 ± 0.43	14.95 ± 2.6
**H460 MN58R**	19.2 ± 2.6 (49)	9.5 ± 1.19 (7)	6.60 ± 1.2 (0.4)
**H460 TCD717R**	28.8 ± 10.5 (73)	10.8 ± 2.26 (8)	4.98 ± 0.9 (0.3)

**Table 3:** Synergistic antitumoral effect of cisplatin and ChoKα inhibitors in vivo. The antitumoral effect of the combination of cisplatin and MN58b or RSM932A/TCD-717 was evaluated in vivo using NSCLC xenografts. H460 cells were injected in nude mice, and when tumor volumes reached 0.1 cm^3^, animals were randomly distributed in groups (*n* = 8) to start treatments according to the schedules described in Material and Methods (indicated as the days treatment was performed for each drug or vehicle (DMSO)). Tumor growth inhibition, mean body weight, and statistical significance are shown. More similar efficacy was observed with the combinations than with much higher concentrations of cisplatin alone, significantly reducing toxicity. * statistical significance *p* < 0.05.

MN58b	Drug	Schedule (Days)	Mean Body Weigth (Day 22)	Tumor Gowth Inhibition (Day 22)	*p*
**Control-1**	Vehicle	1,3,4,5,8,10,11,12,15,17,18,19	23.4		
**cDDP**	cDDP (1 mg/kg)	1,4,8,11,15,18	23	33%	0.3
**MN58b**	MN58b (2 mg/kg)	1,3,5,8,10,12,15,17,19	24.4	35%	0.09
**Sequential**	cDDP (1 mg/kg)	1,4	23.9	67%	0.017 *
	MN58b (2 mg/kg)	8,10,12,15,17,19			
**Concomitant**	cDDP (1 mg/kg)	1,4,8,11,15,18	22.5	66%	0.160 *
	MN58b (2 mg/kg)	1,3,5,8,10,12,15,17,19			
**Control cDDP**	cDDP (4 mg/kg)	1,3,5,8,10,12,15,17,19	17.4 *	69%	0.014 *
**TCD-717**	**Drug**	**Schedule (Days)**	**Mean Body Weigth** **(Day 22)**	**Tumor Gowth Inhibition (Day 22)**	***p***
**Control-2**	Vehicle	1,4,8,11,15,18	20.6		
**cDDP**	cDDP (1 mg/kg)	1,4,8,11,15,18	20.9	24%	0.4
**TCD-717**	TCD717 (2 mg/kg)	1,4,8,11,15,18	19.9	31%	0.16
**Sequential**	cDDP (1 mg/kg)	1,4	21.2	51%	0.03 *
	TCD717 (2 mg/kg)	8,11,15,18			
**Concomitant**	cDDP (1 mg/kg)	1,4,8,11,15,18	20.1	55%	0.04 *
	TCD717 (2 mg/kg)	1,4,8,11,15,18			
